# Nanopriming Action of Microwave-Assisted Biofunctionalized ZnO Nanoparticles to Enhance the Growth under Moisture Stress in *Vigna radiata*

**DOI:** 10.1021/acsomega.3c01329

**Published:** 2023-07-27

**Authors:** Raja Kalimuthu, Kumuthan Meenachi Sellan, Dhivya Antony, Sudhagar Rajaprakasam, Vanniarajan Chokkalingam, Prabu Chidambaram, Selvaraju Kanagarajan

**Affiliations:** † Anbil Dharmalingam Agricultural College & Research Institute, TNAU, Trichy 620027, Tamil Nadu, India; ‡ Department of Nano Science & Technology, TNAU, Coimbatore 641003, India; § Department of Chemistry, Dhanalakshmi Srinivasan Arts and Science (co-education) College (Affiliated to University of Madras), Mamallapuram, Chennai 603104, Tamil Nadu, India; ∥ Plant Breeding and Genetics, Tamil Nadu Agricultural University, TNAU, Coimbatore 641 003, India; ⊥ Department of Environmental Science, Tamil Nadu Agricultural University, Coimbatore 641 003, India; # Department of Plant Breeding, Swedish University of Agricultural Sciences, P.O. Box 190, 234 22 Lomma, Sweden

## Abstract

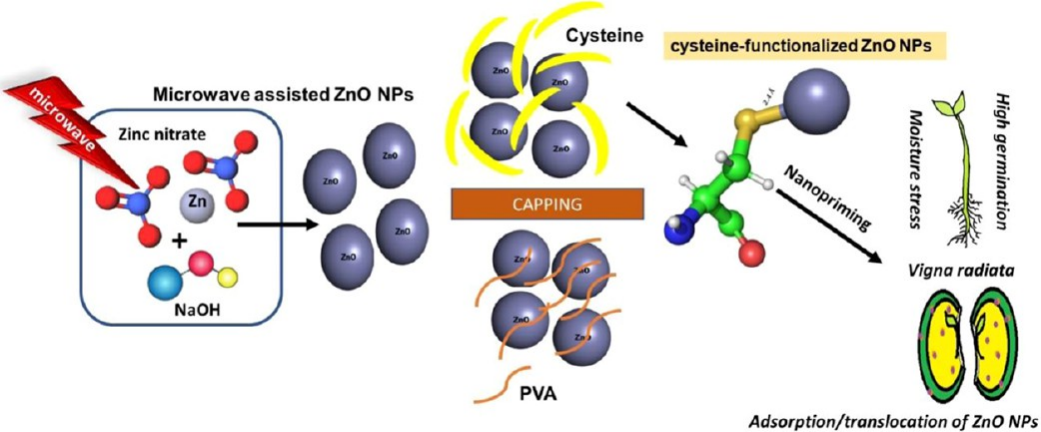

Bare and stabilized zinc oxide nanoparticles (ZnO NPs) were prepared by a microwave-assisted method and used as a priming agent to improve the morphological, physiological, and biochemical quality of *Vigna radiata*. The priming action was made under normal and moisture stress conditions. A microwave reactor of 850 watts power was used to heat 30 mL of a nanocolloidal solution at 140 °C for 20 min. The stable spherical ZnO NPs at 50.4 mV with 28.2 nm particle size were generated and capped with different biomolecules, cysteine and PVA, to get biostabilized ZnO NPs at 48.8 and 108.5 nm with ζ potentials of −56.2 and −52.0 mV, respectively, holding distinct morphology. The nanopriming effect was studied in *V. radiata* seeds for bare ZnO and capped ZnO NPs under normal and moisture stress environments. Cysteine-capped ZnO NPs at 250 ppm showed improved germination (90 and 76%), radicle growth (7.6 and 3.6 cm), seedling Vigor (3064 and 1816), dry matter production (145.06 and 96.92 mg/25 seedlings), and hydrolytic (α-amylase and protease) and antioxidant (peroxidase and superoxide dismutase) enzyme activity under normal and moisture stress conditions. The improved priming action of cysteine-capped ZnO NPs is due to increased cell elongation and cell division in the radicle. The uptake and translocation of ZnO NPs in the *V. radiata* root are evidenced by the presence of an 11.4 ppm zinc level, which was also supported by EDAX and FITC labeling results.

## Introduction

Crop productivity in agriculture depends on the quality of seeds, which can be enhanced by varied techniques to boost germination and seedling vigor.^1^ Nanotechnology is an important problem-solving research area that offers solutions to improve agricultural productivity today. The practice of nanotechnology in agriculture is very new, and few studies have clearly shown the importance of nanoparticles in improving seed quality. Although diverse nanoparticles such as metals and metal oxide NPs, semiconductors, and nanoemulsions have exceptional morphological, structural, and physicochemical properties, these nanoparticles can currently be prepared, analyzed, and used in a wide range of agricultural applications.^2^ Zinc oxide NPs (ZnO NPs), one of the most widely utilized NPs, are often used in various applications, including packaged foods,^3^ biomedical applications,^4−6^ modernizing agriculture,^7^ textile products,^8^ and environmental photocatalysts.^9^ ZnO NPs influenced the growth dynamics, yield, and nutritional composition of black mustard, emphasizing the need for modern stable nanofertilizers to enhance crop values.^10^

Zinc oxide nanoparticles are synthesized mainly by physical and chemical methods, which may cause high costs and loss of energy and be hazardous and time-consuming. The use of microwaves to prepare metal oxide NPs overcomes all these demerits.^11^ The microwave-assisted ZnO nanoparticle synthesis has been the subject of numerous studies, primarily because they offer superior heating rates compared to conventional and volumetric heating.^12^ Also, microwaves typically provide high energy by penetrating the material, allowing the reaction to be finished in a shorter time. Hence, microwave-synthesized ZnO NPs have high purity, affordability, reproducibility, and adherence to environmental criteria.^13^

Assi et al. applied a microwave-assisted sol–gel process to prepare 27 nm sized ZnO NPs with zinc acetate dihydrate as a precursor.^14^ Rose et al. used a microwave-assisted approach to prepare rod-shaped zinc oxide NPs.^15^ Hasanpoor et al. reported ZnO NPs in controlled morphology using a microwave with the increase in time from 10 to 15 min and resulted in the formation of needle-shaped particles with a diameter of 50–150 nm.^16^ The successful fabrication of NPs depends on the size and nanocrystal capping with organic armor to prevent aggregation. The capping process involves the adsorption of organic molecules on the surface of nanoparticles to modify the crystallographic surface energy, encourage the growth of anisotropic nanocrystals, and avert particle aggregation.^17^ According to Bera and Basak, capping ZnO NPs with poly(vinyl alcohol) produced 50 nm sized particles and had a UV–vis spectra peak at 376 nm.^18^ The constant functionalization of biomolecules identified the complex formation of tryptophan-capped ZnO NPs and their binding nature.^19^

The interplay of NPs in plant systems depends on species, seed age, experimental temperature, exposure method, duration, and, more importantly, the size and concentrations of nanoparticles. The germination and seedling vigor are boosted with different bio-capped NP priming methods.^20,21^ Several metal-based NPs and carbon-based NPs have increased the quality of seed properties related to structural, functional, and molecular alterations in recent years.^22,23^ When a seed is infused with NPs or nanoparticles, the metabolic process is triggered, resulting in increased germination, seedling vigor, and plant growth. Numerous studies demonstrated that seed priming with metal and metal oxide NPs could enhance biochemical and molecular traits such as improved enzymatic hydrolysis, food material metabolism, hormone actions, antioxidant capabilities, chromosomal stability, and cell growth.^24,25^ The seed yield was increased by ZnO NPs and Zn^2+^ compounds on different dosages from 160 mg Zn/kg to 400 mg Zn/kg, inducing the least oxidative stress. ZnO NPs could be an innovative nanofertilizer to add Zn to the required soil.^26^ According to Francis et al., ZnO NPs on plants at 0.12 and 0.24 M exhibit significant antioxidant concentrations, ensuring better plant protection from diseases and pests and boosting plant growth.^27^ To improve plant and Zn nutrient growth for agricultural sustainability, Itroutwar et al. synthesized biogenic ZnO NPs as an efficient nanopriming agent for seed treatment.^28^

Generally, the seed goes through many metabolic changes throughout the germination period. The in vitro bio-efficacy investigation of PVA nanofiber-coated seeds exhibited better germination rates, root and shoot length, seedling vigor, dry matter production, plant biomass, root volume, nodule counts, and fresh nodule weight.^29^ The seed priming with capped ZnO NPs significantly enhances both germination and seedling vigor, which is decided by the specified concentration, duration, and treatment method.^30^ To promote NP stability by avoiding agglomeration with enhanced properties and bioavailability, the current work modified the surface of ZnO NPs with cysteine and PVA as a capping organic layer. *Vigna radiata* L. (green gram or mung bean) was used to test the prepared nanoparticle’s bio-efficacy in normal and moisture-stressed settings. Green gram is a pulse crop that accounts for major sources of dietary protein in the vegetarian diet in India and is mainly cultivated under a rainfed ecosystem (>70% area) where poor germination and crop establishment are the common constraints causing productivity loss.^31^ A viable and promising seed invigoration is essential to overcome these problems. Hence, the research focused on the green gram.

## Materials and Methods

Analytical-grade zinc nitrate (98%), sodium hydroxide (99%), cysteine (97%), and PVA (99%) were purchased from Merck. Genetically pure seeds of the green gram variety CO 8 were used, which were procured from the Department of Pulses, TNAU, Coimbatore. The seeds were washed, dried, and size-graded using an 8×8 BSS sieve before being evaluated in a lab for initial seed quality.

### Synthesis of ZnO NPs

4.54 g of zinc nitrate (1.6 mol L^–1^) in 15 mL was made up to 32 mL with deionized water to get a Zn^+2^ solution. After that, a colloid of ZnO NPs was obtained by adding 4 mL of 0.5 g of sodium hydroxide (3.2 mol L^–1^) dropwise into the Zn^+2^ solution with steady magnetic stirring at room temperature for 10 min. The reaction mixture was then put into a glass vial measuring 30 mL and heated to 140 °C under temperature control for 20 min in a microwave reactor (Anton Paar Monowave 450) at 850 W. The reacted mixture was cooled to room temperature, and the white precipitate that had gathered at the bottom of the vial was filtered and washed thrice with deionized water and ethanol. The product was dried for 3 h at 65 °C in a vacuum oven.^32^

### Functionalization of ZnO NPs

Cysteine and PVA molecules capped ZnO NPs separately by utilizing nanocapping methods.^33,34^ To prepare cysteine-capped ZnO NPs, 0.82 mmol of cysteine was dissolved in 10 mL of deionized water, and 1 mol of NH_4_OH was used to bring the pH down to 9. Cysteine was highly protonated at pH 9 with its side chain sulfhydryl (−SH) group, which promoted the best binding on ZnO NPs. The cysteine solution was then added to 50 mg of ZnO NPs that had been synthesized using a microwave, dispersed for 10 min, filtered, and then dried at room temperature.

PVA-capped ZnO NPs were prepared by adding 0.05 g of poly(vinyl alcohol) dissolved in 50 mL of deionized water with 4 g of ZnO NPs on vigorous stirring at 50 °C. Then, pH 12 was maintained by adding 10 mL of a 1% NH_4_OH solution to sustain the viscosity and enhance PVA’s capping power on ZnO NPs. The white precipitate was filtered and repeatedly rinsed with deionized water and then dried in an oven at 90–100 °C. The change in pH took relevance to the size and morphology of ZnO NPs, which impacted distinct properties and enhanced the application efficacy. This transformation in functionalized ZnO NPs by cysteine and PVA was further characterized, and the best-functionalized ZnO NPs were applied in nanopriming *V. radiata* seeds.

### Characterization Techniques

ZnO NPs were characterized using a Horiba Scientific NPs SZ-100 (NPs analyzer) to find particle size and ζ potential. UV–visible spectroscopy (UV–vis) was used to determine the absorbance and wavelength in the range of 100–400 nm. Raman spectroscopy was employed to identify the composition of NPs based on the inelastic scattering method. a RENISHAW confocal Raman microscope (United Kingdom) with a 532 nm excitation laser source (50 mW) was used to get the clear symmetry surface structure and molecular bonding of ZnO NPs. The XRD pattern was recorded by a Cu K radiation width of 1.5406, and the scanning was carried out for the range of 0–100 at a speed of 5° per min at room temperature (25 °C). Fourier transform infrared (FT-IR) spectra for synthesized ZnO NPs were obtained using a JASCO FTIP/6800 spectrometer (JASCO Japan) equipped with an attenuated total reflectance unit (ATR) sensor. Spectral data between 400 and 4000 cm^–1^ were collected, averaging 64 scans at a resolution of 4 cm^–1^.

A scanning electron microscope (SEM FEI QUANTA 250) with EDAX was used to characterize the size, morphology, and elemental composition of the synthesized ZnO NPs. A sample of test NPs (0.5–1.0 mg) was dusted on one side of the double-sided adhesive carbon conducting tape mounted on the 12 mm aluminum stub. The sample surface was observed at different magnifications, and the images were recorded. A transmission electron microscope (TEM FEI Technai Spirit) was used to analyze the diluted suspensions of synthesized ZnO NPs in pure ethanol by ultrasonication. A drop of the suspension was placed on a 300-mesh lacy carbon-coated copper grid and dried, and the images were captured at different magnifications.

### Nanoseed Priming Effect in Optimal and Moisture Stress Conditions

The optimized level of 250 ppm ZnO NPs was chosen for nanopriming after analyzing with 10–500 ppm ZnO NPs on *V. radiata* substandard seeds. Rapid germination with the high active enzymatic result was shown at 250 ppm without damaging the seeds. The nanopriming result was obtained by comparing untreated seeds (control) in normal and moisture stress conditions. To restore the innate moisture content of the green gram, hereditarily and physically pure seeds were soaked for 3 h according to the treatment like T1—untreated seeds, T2—hydropriming, T3—cysteine @ 250 ppm, T4—PVA @ 250 ppm, T5—ZnO NPs @ 250 ppm, T6—cysteine-capped ZnO NPs @ 250 ppm, and T7—PVA-capped ZnO NPs @ 250 ppm. After the treatments, the seeds were dried in the shade. The observations of the change in radicle growth, germination percentage, root length, shoot length, dry matter production, vigor index, enzyme activity (α-amylase, protease, peroxidase (POD), and superoxide dismutase (SOD)) were obtained under normal and moisture stress environments. All of the growth parameters were noted in moisture stress conditions by 6000 MW poly(ethylene glycol) (PEG). After being primed with ZnO NPs, various levels of moisture stress were noted by applying 10, 20, 30, and 40 gm of PEG dissolved in a liter of deionized water under osmotic potentials of 1.5, 4.9, 10.3, and 17.6 eV. Under normal and stressful circumstances, treated and untreated seeds were examined for changes in seed quality in an in vitro environment. Four replications of the experiment were conducted using a completely random design (CRD).

An equal amount of treated and untreated seeds was sowed in Petri plates, included with two layers of humid germination paper, and placed in natural lighting. After sowing, the radicle growth was gauged at 24, 48, and 72 h. The radicle length was measured on 10 germinated seeds (radicle growth should be greater than or equal to 0.5 mm) from each treatment replication. The roll towel method was conducted with 25 seeds for each treated in the germination room under the test conditions of 25 °C and 95° RH. After 7 days, the mean germination rate was calculated and expressed as a percentage. Ten healthy seedlings were randomly selected from the conventional germination test on the last day to assess the root length. The major root’s tip was measured at the collar area of the root, and the shoot length was measured from the collar area to the growing tip of the shoot. The mean value of the root and the shoot length was calculated in centimeters. The seedlings used for measuring growth were shade-dried for 24 h, followed by another 24 h of drying in a hot air oven maintained at 85 °C, and finally, 30 min of cooling in a silica gel desiccator. Using an electronic balance, the dry weight of the seedlings was calculated in milligrams. The Abdul-Baki and Anderson approach was used to calculate the vigor index, which was then expressed as a whole number.^35^
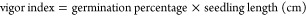


### Hydrolytic Enzyme Activity

To extract the enzymes for α-amylase activity, 500 mg of pregerminated seeds from each treatment and replication were weighed, homogenized in 1.8 mL of cold 0.02 M sodium phosphate buffer (pH 6.0), and centrifuged at 20,000 rpm for 20 min. One milliliter of a 0.067 percent starch solution was added to 0.1 mL of enzyme extract. After 10 min of incubation at 25 °C, the reaction was halted by adding 1 mL of iodine HCl solution (60 mg of KI and 6 mg of I_2_ dissolved in 100 mL of 0.05 N HCl). A double-beam spectrophotometer was used to measure the color change at 620 nm. The mean values were represented in mg maltose min^–1^, and the amylase activity was determined using the following formula.
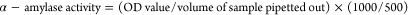
In a prechilled pestle and mortar, 200 mg of pregerminated seeds were homogenized with 10 mL of 0.2 M sodium phosphate buffer to measure protease activity. The homogenate was centrifuged in a chilled centrifuge at 4 °C for 15 min at 15,000 rpm. 0.35 mL of 0.5% casein was added to 1 mL of the supernatant, which was then incubated at 37 °C for 1 h. The reaction was then halted by adding 2 mL of 10% ice-cold trichloroacetic acid (TCA). A reaction combination made up of 2.5 mL of sodium acetate buffer, 0.75 mL of Folin reagent, and 1 mL of distilled water to receive 1 mL of the supernatant from this mixture. After 20 min of incubation at 37 °C, the protease activity was measured by the difference in absorbance at 660 between control and tested samples.^36^
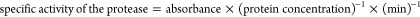


### Antioxidant Enzyme Activity

Sodium phosphate buffer was used to extract the peroxidase from the leaves of seedlings at 10 days for each replicated treatment outlined as follows. The reaction mixture comprised 2.5 mL of a solution containing 0.25% (v/v) guaiacol, 0.01 M sodium phosphate buffer, pH 6.0, and 0.1 M hydrogen peroxide. The reaction was started by adding 0.1 mL of the enzyme extract and monitored calorimetrically at 470 nm. To achieve changes in absorbance at 470 nm of 0.1–0.2 absorbance units/min, crude enzyme preparations were diluted. The heated enzyme served as the control. The increase in absorbance at 420 nm of seedling content was used to measure activity.^37^ 500 mg of seed samples were macerated in 10 mL of 0.2 M phosphate buffer to create the crude enzyme extract to measure the superoxide dismutase (SOD) activity. 50 mL of the crude enzyme extract was added to 2.95 mL of the reaction medium, which contained 1.5 mL of 50 mM sodium phosphate at pH 7.8. 0.2 mL of 13 mM methionine, 0.1 mL of 75 mM nitro blue tetrazolium (NBT), 0.1 mL of 0.1 mM EDTA, and 0.1 mL of 2 mM riboflavin taken in a reaction chamber with a 15 W fluorescent bulb, and the reaction was conducted at a temperature of 25 °C. Blue formazan, obtained by the photoreduction of NBT, was determined by absorbance at 560 nm after 5 min of exposure to light.^38^

### SEM Analysis with EDAX for the Ultramicrotome-Partitioned Radicle

The topographical changes in the radicle cell of the green gram seed were observed by SEM with EDAX for the ultramicrotome-partitioned radicle. The best-resulting cysteine-capped ZnO NP-primed seed was used along with untreated seeds to detect the adsorption, absorption, and translocation of ZnO NPs in the radicle, seed coat, cotyledon, and embryo axis under optimal and moisture stress. After 24 h of imbibition of ZnO NPs on the sprouting stage, the radicle growth was noticed and trimmed. The sliced radicle was sectioned using an ultramicrotome and inspected using a scanning electron microscope (SEM), and the elemental Zn percent was noted through EDAX.

### Fluorescent Observation of FITC-Conjugated ZnO NPs in Radicles

The cysteine-capped ZnO NPs were conjugated for 8 h in the dark with 5 μL/mL fluorescein isothiocyanate (FITC). Green gram seeds were soaked in FITC-conjugated ZnO NP solutions set at 18 °C with 16/8 h light/dark cycles and 2000 lux of light intensity in the growth chamber. The soaked seeds and untreated ones started to sprout in the Petri plates. Radicles were sliced and washed with phosphate-buffered saline (PBS) solution after being ingested for 24 h. Following buffer washing, a small piece of the radicle was fixed for 6 h in 4% paraformaldehyde. Then, the radicle and plumule were imaged using a fluorescence microscope equipped with a cooled CCD camera. The cysteine-capped ZnO NP-primed seed and the untreated seed radicle were sectioned and stained with 3% uranyl-acetate to predict TEM images. The translocation of Zn as a result of ZnO NP priming was analyzed using an X-ray fluorescence (XRF) measuring system. The arithmetical data from various trials were evaluated using the statistical methods of agricultural research.^39^ At a 5% probability level, the crucial differences (CDs) were determined. The percentage-based data were converted into angular values (arcsine transformation).^40^

## Results and Discussion

The microwave-synthesized ZnO NPs were first prepared and later capped with cysteine and PVA molecules. ZnO NPs with high surface energy possess more aggregation, and functionalization techniques can prevent this. Herein, innovative functionalized ZnO NPs with PVA and cysteine are prepared in simple mode and compared with pure ZnO NPs. The conjugated and monodispersed ZnO NPs improve characteristic properties and enhance the nanopriming effect on seeds. The functionalized ZnO NPs act as a capping agent with carboxyl and thiol groups, which hold water solubility and size stability possessing a high ability to bind with phytopigments in seeds. Cysteine-functionalized ZnO NPs were highly effective in nanopriming, and they were chosen based on their functional group support in enzymatic action in plant tissues. Imposing cysteine-functionalized ZnO NPs in priming was the highlight, and advancement in seedling’s growth was identified with its results.

**Figure 1:**
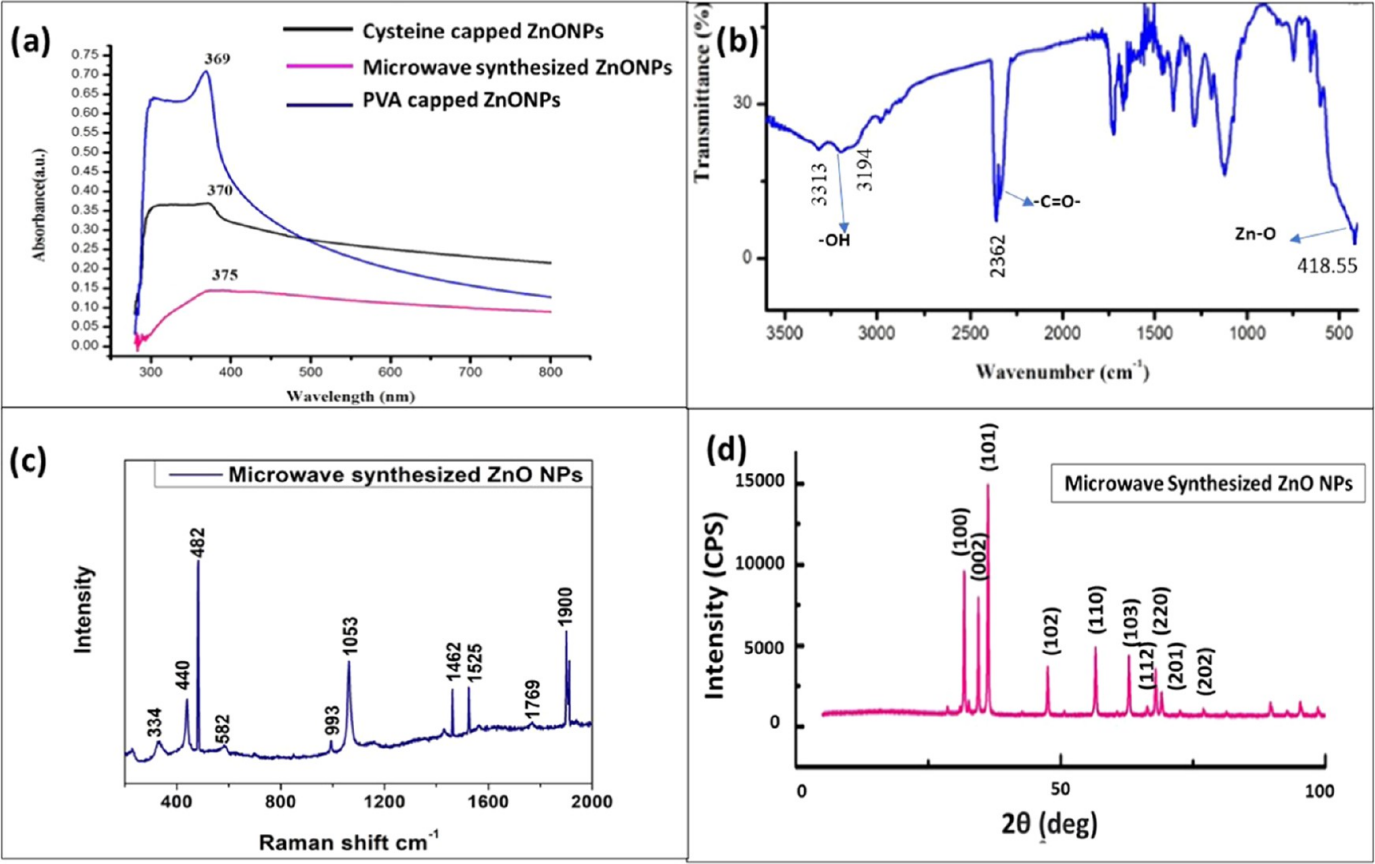
Microwave-synthesized ZnO NPs: (a) UV–vis spectrum, (b) FT-IR spectrum, (c) Raman spectrum, and (d) XRD pattern.

Microwave-synthesized ZnO NPs had a size of 28.2 nm using a particle size analyzer, and its ζ potential was 50.4 mV indicating the stability of the particles.^41^ In UV–vis, the absorbance peak was noted at 375 nm (Figure 1a). The amino acid cysteine stabilizes ZnO NPs, prevents agglomeration, and gives precise size on clear dispersion. In the Burstein–Moss effect, the ZnO NPs drop as the cysteine concentration increases, resulting in a blue shift of around 35 nm.^33^ The ZnO NP surface and the crystal growth process were modified by the sol–gel technique and in situ coated with amino acid cysteine. The density of surface oxygen deficiency hierarchical nanostructures was lowered by chelating hydrophilic thiol-like cysteine to modulate the optical emission and stability.^42^ The high surface charge of ZnO NPs causes the particles to aggregate, which by adding an appropriate ligand develops the surface coating and improves colloidal stability.^43^ In the literature, the capped ZnO NPs show a shift in absorbance and wavelength value, showing that cysteine and PVA get adsorbed on the ZnO NP surface with modified size.

FT-IR spectra showed a broadband between 800 and 410 cm^–1^, which is a characteristic transmittance peak range of the Zn–O bond noted at 418.55 cm^–1^ for synthesized ZnO NPs.^22,23^ The presence of the functional group in ZnO NPs is shown in Figure 1b; C=O was noted at 2362 cm^–1^and −OH was observed at 3313 and 3194 cm^–1^, where the metal oxide frequency was noted at 418.55 cm^–1^. Microwave-assisted ZnO NPs reported by Ambrozic et al. show peaks at 3313, 3194, and 2362 cm^–1^, indicating the presence of −OH and C=O residues, probably due to atmospheric moisture and CO_2_, respectively, in the KBr matrix.^44^ The microwave-synthesized ZnO NPs yielded the most intense Raman shifts at 482 cm^–1^, which shows a higher shift than its theoretical values.^45^ The main dominant, sharp peaks labeled as E_1_ and E_2_ at 440 and 482 cm^–1^ are observed in Figure 1c. The Raman active optical phonon mode at 440 and 482 cm^–1^ shows the characteristic peaks of wurtzite hexagonal phase ZnO.^46^

**Figure 2:**
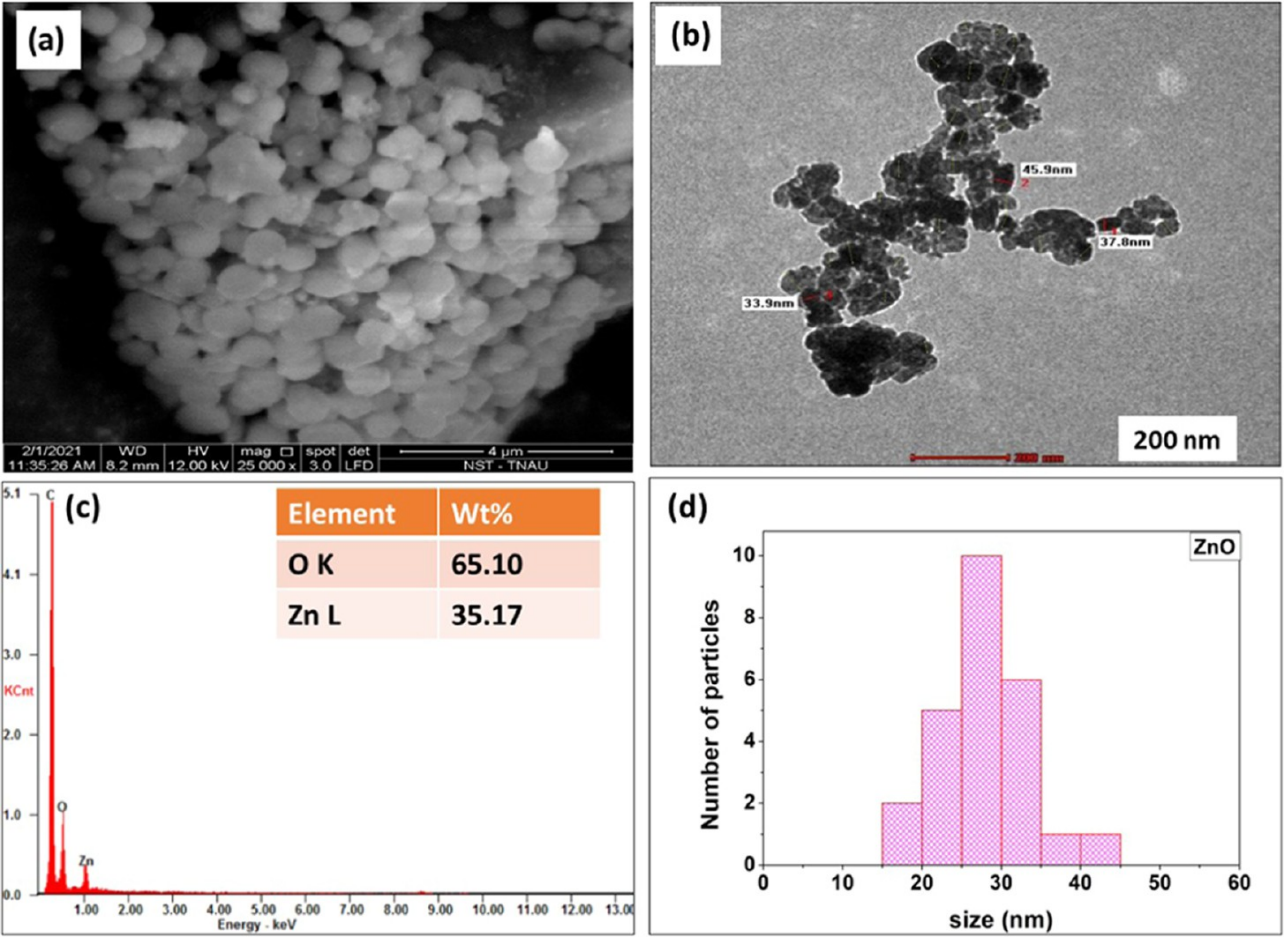
Microwave-synthesized ZnO NPs: (a) SEM images, (b) TEM images, (c) EDAX pattern, (d) histogram.

XRD results of Alnarabiji et al. reported microwave-assisted ZnO NPs, showing strong diffraction patterns at 2θ = 31.8, 34.3, and 36.5°.^47^ Rana et al. observed a diffraction peak at 34.4° for MW-ZnO NPs, which matches with our crystalline nature ZnO NPs having strong diffraction peaks at 31.7, 34.4, 36.3, 47.5, 56.5, 62.8, and 69.0°.^48^ The maximum peak at 36.3° holds a *d*-spacing of 2.47 Å and its crystalline size is noted as 45.6 nm using the Scherrer equation *D* = *k*λ/β·cos θ. In Figure 1b–d, the characterized results of UV–vis, FT-IR, and XRD were predicted to validate the formation of ZnO NPs. Figure 2a shows the SEM images of ZnO NPs in spherical morphology. The TEM images show the average size range from 25 to 30 nm, as shown in Figure 2b. The EDAX spectra in Figure 2c confirmed the elemental composition of zinc as 35% and oxygen as 65%.

### Characterization of Functionalized ZnO NPs

The microwave-synthesized ZnO NPs were capped with cysteine and PVA. They were examined for their physical and chemical characteristics to prevent agglomeration, improve stability with the desired particle morphology and size, and boost efficiency. For cysteine and PVA, the capped nano ZnO displayed particle sizes of 48.8 and 108.5 nm and ζ potentials of −56.2 and −52.0 mV, respectively. PVA capping decreased the aggregation of NPs, which may improve the stability and homogeneity of the particle distribution in the aquatic solution.^34^ UV–visible spectra validate the cysteine- and PVA-capped ZnO NPs, displaying peaks at 370 and 369 nm, respectively. The UV–vis absorbance peak for ZnO NPs with cysteine caps was observed by Deng et al. at 378 nm, confirming the effective capping of cysteine amino acid over the surface of the NPs.^49^

**Figure 3:**
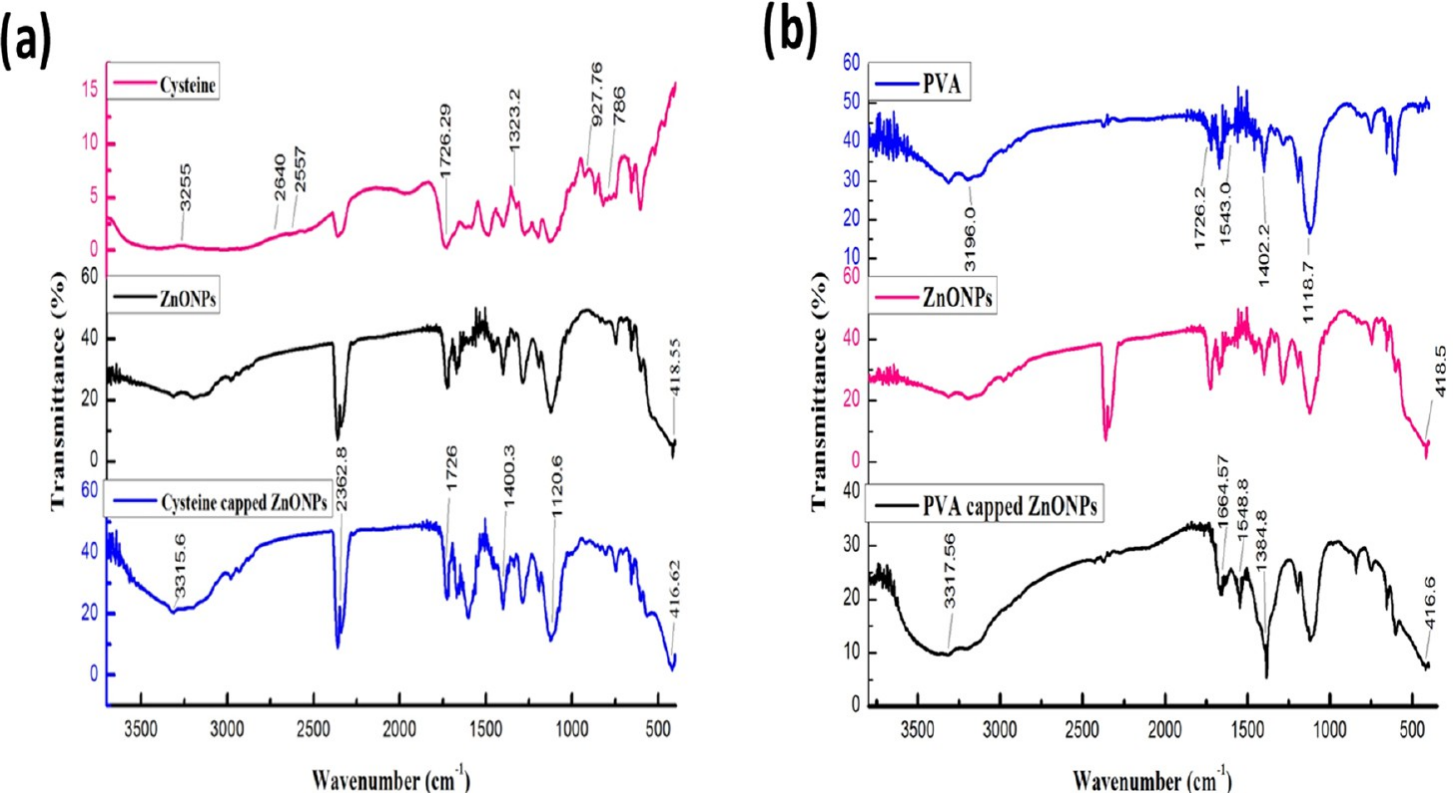
FT-IR spectra: (a) cysteine, ZnO NPs, and cysteine-capped ZnO NPs and (b) PVA, ZnO NPs, and PVA-capped ZnO NPs.

FT-IR analysis of pure cysteine revealed strong bands at 2362 cm^–1^ (SH str. asymmetry), 1726 cm^–1^ (C=O), 1581 cm^–1^ (NH3 bend), 1483 cm^–1^ (N=O str), 1323 cm^–1^ (NH3 bend asymmetry), 1274 and 1197 cm^–1^ (CH2), 1122 cm^–1^ (NH3 twist), 927 cm^–1^ (SH bend), and 819 cm^–1^ (COO wagg.). Figure 3a shows the FT-IR spectra of cysteine-capped ZnO NPs, with spectral peaks at 1517 and 1602 cm^–1^, confirming the carboxylic and carbonyl group (C=O) interaction of cysteine with the ZnO surface. Further, the peak appeared at 2527 cm^–1^ in cysteine and was found to be depressed in cysteine-capped ZnO NPs, which also strongly indicated the interaction of the thiol group with ZnO NPs. The FT-IR peak for cysteine at 2540 cm^–1^ was suppressed in cysteine-capped ZnO NPs, confirming the interaction of thiol groups with ZnO NPs.^50^ In Figure 3b, the PVA-capped ZnO NPs showed the transmittance peaks at 3317.56, 1664.57, 1548.84, 1384.89, and 416.62 cm^–1^ for confirming the functional group of both capping agents as well as ZnO NPs. The C=O (str.) band at 1726 cm^–1^ in PVA shifted to a lower wavenumber in the ZnO–PVA nanocomposite, which indicated and confirmed the interaction between zinc oxide and PVA.

**Figure 4:**
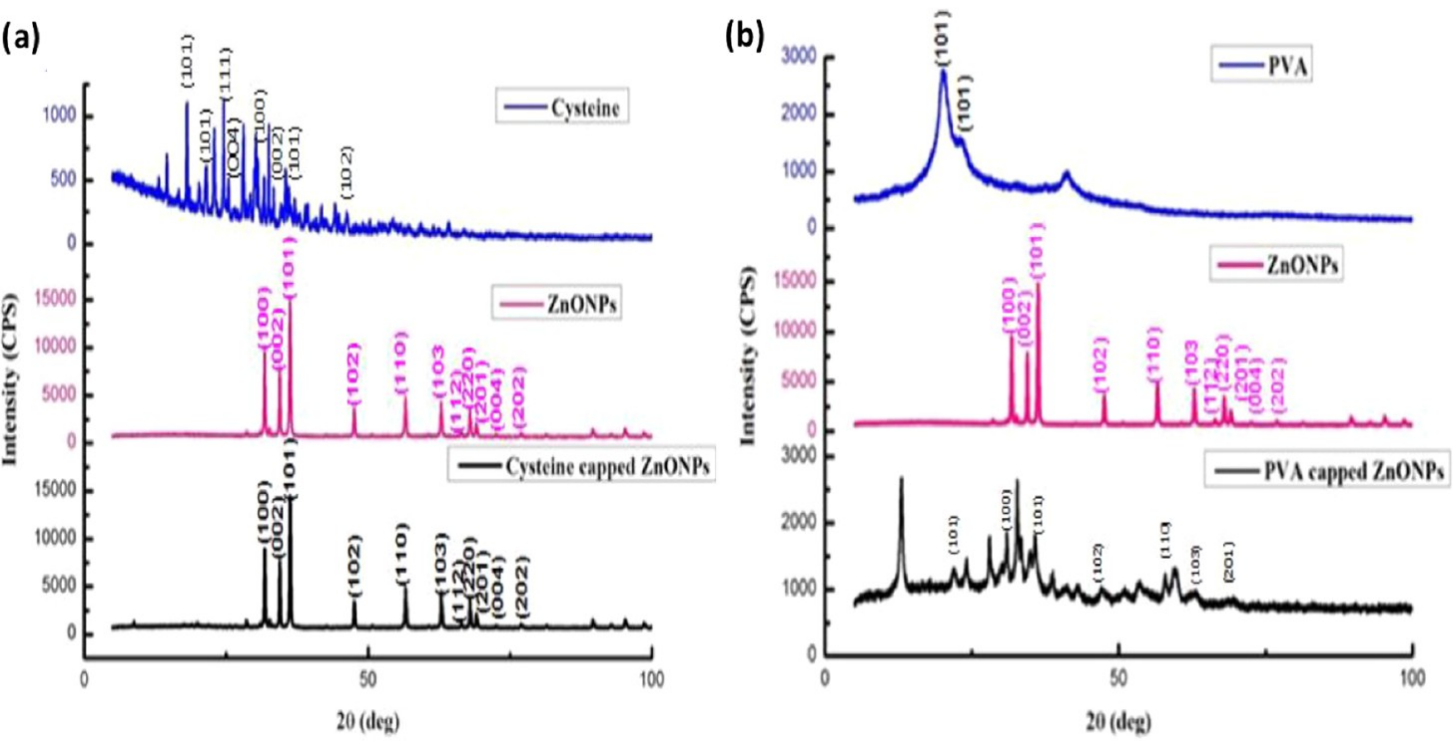
XRD spectra: (a) cysteine, ZnO NPs, and cysteine-capped ZnO NPs and (b) PVA, ZnO NPs, and PVA-capped ZnO NPs.

Figure 4a shows the strong diffraction peaks observed at 19.8, 31.7, 34.43, 36.2, 47.5, 56.6, 62.8, and 67.9° for the cysteine-capped ZnO NPs, displaying the largest peak at 36.2° with a *d*-spacing of 2.47 Å, while PVA-capped ZnO produced strong diffraction peaks at 12.9, 24.0, 30.9, 32.6, 33.3, 34.9, 35.6, and 59.4°, in which the greatest peak was seen at 32.6° with a corresponding *d*-spacing of 2.74 Å (Figure 4b). The results have strongly revealed the crystallinity in cysteine- and PVA-capped ZnO NPs, with little deviation in PVA-ZnO NPs, without compromising their crystalline nature. Guglieri and Chaboy measured XRD diffraction peaks at 26, 31.2, and 51.7, to report the crystallinity of ZnO NPs with cysteine caps.^51^ Dutta et al. reported on poly(ethylene glycol)-capped ZnO NPs with hexagonal crystalline particles at 9 nm and the UV–vis absorption peak at 355 nm with the broadened XRD pattern.^52^

**Figure 5:**
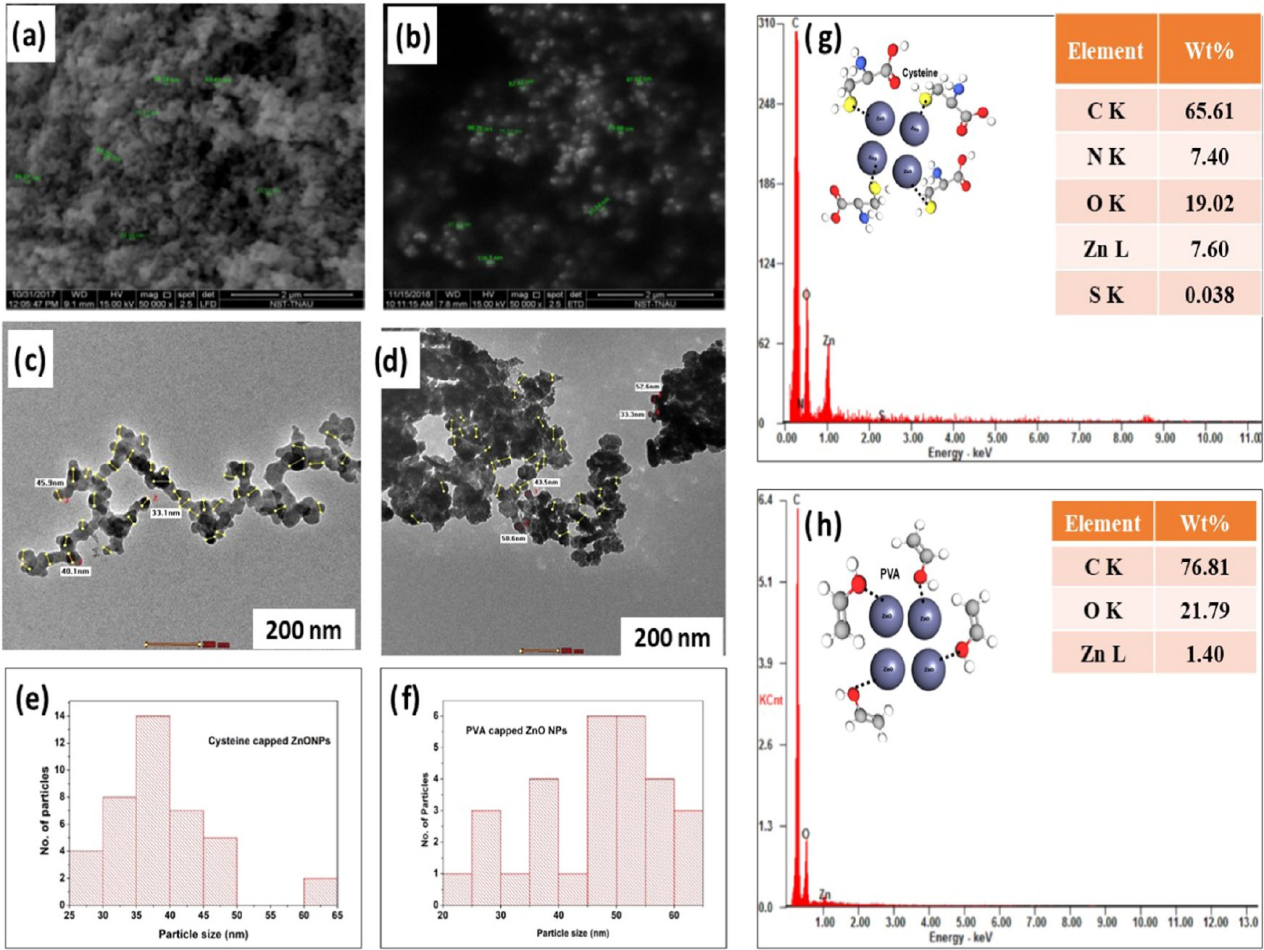
SEM images: (a) cysteine-capped ZnO NPs and (b) PVA-capped ZnO NPs. TEM images: (c) cysteine-capped ZnO NPs and (d) PVA-capped ZnO NPs. (e) Histogram of cysteine-capped ZnO NPs. (f) Histogram of PVA-capped ZnO NPs. (g) EDAX pattern of cysteine-capped ZnO NPs. (h) EDAX pattern of PVA-capped ZnO NPs.

SEM images of cysteine- and PVA-capped ZnO NPs show spherical morphology (Figure 5a,b). The TEM images of cysteine- and PVA-capped ZnO NPs with size ranges of 35–40 and 45–55 nm, respectively, are picturized in Figure 5c,d. The particle sizes in each TEM image were statistically predicted using a histogram, as shown in Figure 5e,f. The elemental composition of the relevant capped ZnO NPs was predicted in EDAX, providing additional support for these findings shown in Figure 5g,h. According to Arslan and Singh, the spherical morphology of cysteine-capped ZnO NPs ranged in size from 5 to 15 nm.^42^

The electrostatic and hydrophobic interactions govern the structure of cysteine and PVA to compensate for the stability of ZnO NPs. The interaction of cysteine biomolecules gets affinity on ZnO NPs with their thiol group (−SH), showing the best interaction on the surface of ZnO NPs. But in PVA, the attraction is made by the oxygen atom to get bound on the ZnO NP surface. The stability of cysteine-functionalized ZnO NPs is highly stable compared to PVA-functionalized ZnO NPs due to the nature of interaction compelled by the biomolecules. The biomolecules on electrostatic interactions constrain their conformations on the surface of ZnO NPs, and their arrangement is picturized and inserted in Figure 5g,h. The cysteine-capped ZnO NPs have high stability and the best reactivity due to the presence of thiol groups.^52^

### Responses of Green Gram Seeds Exposed to ZnO NPs

The optimized phytotoxic level of ZnO NPs at 250 ppm was used to priming seeds. To monitor the parameter changes in seed nanopriming, treatments were made by T1—untreated seeds, T2—hydropriming, T3—cysteine @ 250 ppm, T4—PVA @ 250 ppm, T5—ZnO NPs @ 250 ppm, T6—cysteine-capped ZnO NPs @ 250 ppm, and T7—PVA-capped ZnO NPs @ 250 ppm dramatically altered the radicle development of green gram. Variation was noted in all treated and untreated seeds, and then the accumulation of Zn in seedlings was tested by imaging the segmented shoot and root parts with TEM and EDAX. *V. radiata* seeds under moisture stress conditions were studied with cysteine-capped ZnO NPs at 250 ppm, showing the best result.

**Figure 6:**
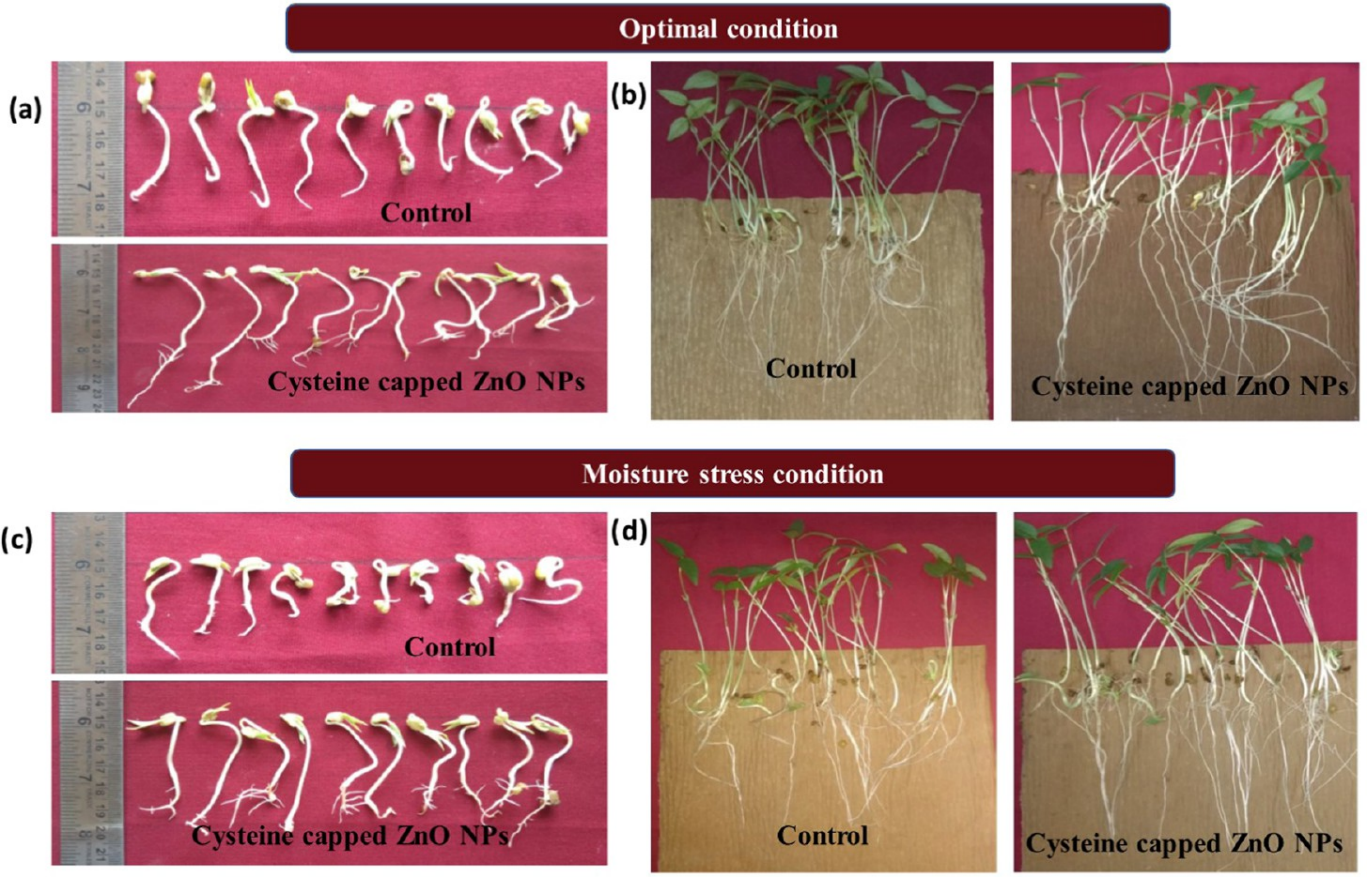
(a) Radicle sprout of nanoprimed green gram in optimal conditions. (b) Seedling growth of nanoprimed green gram in optimal conditions. (c) Radicle sprout of nanoprimed green gram in moisture stress conditions. (d) Seedling growth of nanoprimed green gram in moisture stress conditions.

In an in vitro setting, primed and untreated seeds were tested for changes in seed quality under optimal and moisture stress conditions. All of the experiments were replicated four times using an entirely random design (CRD). Implementing Panse and Sukhatme strategies, the data generated in our experiments were statistically analyzed. At a 5% probability level, the crucial differences (CDs) were determined, and percentage-based statistics were converted into angular values using the arcsine transformation. Under optimal and moisture stress conditions, seed invigoration with nanoparticles considerably alters the morphological, physiological, and biochemical seed quality parameters. The best radicle growth of cysteine-capped ZnO NP-primed green gram cv. CO 8 under optimal and PEG moisture stress conditions is shown in (Figure 6a,c) compared to their controls. Seed quality parameters and the enzymatic activities of green gram under optimum conditions are presented in Table 1. In normal conditions, seeds primed with 250 ppm cysteine-capped ZnO nanoparticles accelerated radicle growth, resulting in 77.6 and 51.0% increases in 48 h. The moisture stress of cysteine-capped ZnO NP-primed seeds improves the radicle development rates up to 77.0 and 44% compared with ZnO NPs and untreated seeds, respectively. ZnO nanopriming significantly showed radicle growth 48 h after seeding, even at a high moisture stress of 17.6 bar. The longest radicle of 3.4 cm was formed by seeds primed with cysteine-capped ZnO NPs at a concentration of 250 ppm, where ZnO NPs showed 2.1 cm and control showed 1.6 cm. In Table 2, all growth parameters were recorded under normal and moisture stress (PEG) conditions. Figure 6b,d illustrates the best seedling growth for the cysteine-capped ZnO NP-primed green gram cv. CO 8 under optimal conditions and PEG-induced moisture stress conditions are shown with the comparison of untreated seeds. Table 3 and 4 shows the correlation among the seed quality parameter in nano-primed seeds of cv. CO 8 under optimal condition and PEG-induced moisture stress.

**Table 1:** Nanopriming on Seed Quality Parameters in Green Gram cv. CO 8 under Optimum Conditions^a^

	radicle growth (cm)								enzyme activity		antioxidant activity	
treatment	24 h	48 h	72 h	germination (%)	shoot length (cm)	root length (cm)	vigor index	dry matter (mg/25 seedlings)	α-amylase activity (mg maltose min^–1^)	protease activity (U mg^–1^ of protein min^–1^)	peroxidase activity (min^–1^ mg^–1^ of tissue)	superoxide dismutase activity (U mg^–1^ of protein)
T1	1.4	2.3	4.2	72 (58.03)	11.3	11.7	1681	99.74	0.439	0.0011	0.857	7.19
T2	1.5	2.4	4.3	76 (61.25)	11.7	12.1	1773	100.5	0.497	0.0012	1.07	8.2
T3	1.7	3.1	5.1	78 (62.86)	14.6	14.9	2299	110.82	0.592	0.0017	1.608	11.9
T4	1.3	2.4	4.2	73 (58.83)	11.9	13.9	1888	111.19	0.503	0.0014	1.23	9.13
T5	1.7	3.3	5.8	81 (65.28)	14.2	15.5	2405	126.54	0.644	0.0018	1.64	13.31
T6	2	4.1	7.6	90 (72.54)	15.9	18.1	3064	145.06	0.932	0.0021	2.09	16.2
T7	1.8	3.6	6.6	81 (65.28)	13.6	16.4	2434	128.25	0.704	0.0019	1.93	14.1
mean	1.6	3.0	5.4	64.18	13.3	14.7	2220.6	117.4	0.6	0.0016	1.5	11.4
SEd	0.054	0.104	0.103	0.782	0.512	0.432	24.23	2.351	0.0102	0.0009	0.0153	0.1325
CD (*P* = 0.05)	0.107	0.206	0.204	1.564	0.104	0.853	48.46	4.702	0.0203	0.0018	0.0226	0.2643

a Treatments T1—untreated seeds, T2—hydropriming, T3—cysteine @ 250 ppm, T4—PVA @ 250 ppm, T5—ZnO NPs @ 250 ppm, T6—cysteine-capped ZnO NPs @ 250 ppm, and T7—PVA-capped ZnO NPs @ 250 ppm.

**Table 2:** Nanopriming on Seed Quality Parameters in Green Gram cv. CO 8 under PEG-Induced Moisture Stress Conditions^a^

							enzyme activity		antioxidant activity	
treatment	radicle growth (cm)	germination (%)	shoot length (cm)	root length (cm)	vigor index	dry matter (mg/25 seedlings)	α-amylase activity (mg maltose min^–1^)	protease activity (U mg^–1^ of protein min^–1^)	peroxidase activity (min^–1^ mg^–1^ of tissue)	superoxide dismutase (U mg^–1^ of protein)
T1	1.9	59 (47.55)	6.4	6.8	662	46.43	0.319	0.001	0.687	5.69
T2	2.1	63 (50.79)	8.8	6.9	896	59.32	0.377	0.0011	0.9	6.69
T3	2.9	67 (54.00)	11.9	8.1	1126	73.05	0.472	0.0016	1.438	10.39
T4	2	59 (47.55)	6.9	5.9	662	46.43	0.383	0.0013	1.06	7.62
T5	2.8	71 (57.22)	10.8	8.3	1233	72.85	0.524	0.0016	1.47	11.82
T6	3.6	79 (63.67)	14.4	10.1	1816	96.92	0.812	0.002	1.92	14.69
T7	3	67 (54.00)	10.9	8.9	1210	75.72	0.584	0.0018	1.76	12.59
mean	2.6	64.4 (51.06)	10.0	7.9	1086.4	67.2	0.5	0.0	1.3	9.9
SEd	0.053	1.523	0.406	0.234	52.13	2.86	0.203	0.00002	0.281	0.186
CD (*P* = 0.05)	0.106	3.105	0.812	0.468	104.26	5.72	0.407	0.00004	0.564	0.376

a Treatments: T1—untreated seeds, T2—hydropriming, T3—cysteine @ 250 ppm, T4—PVA @ 250 ppm, T5—ZnO NPs @ 250 ppm, T6—cysteine-capped ZnO NPs @ 250 ppm, and T7— PVA-capped ZnO NPs @ 250 ppm.

In optimal and moisture stress conditions, priming with cysteine-ZnO NPs records higher levels of the antioxidant enzymes peroxidase and superoxide dismutase due to decreased ROS-induced oxidative damage and maintained cell membrane integrity. Seed priming with 20 mg/L ZnO NPs capped with phytogroups represents the enhanced germination rate, starch metabolic process, and triggered zinc acquisition of rice-aged seeds.^30^ The phytochemical-coated Ag NPs prepared by Mahakham et al. served as a nanocarrier and increased the activity of α-amylase in primed maize seeds, which converted starch into simple soluble sugar and ultimately the energy as ATP to the growing embryo.^53^ The strong binding of amylase’s active site cysteine thiol group-coated NPs increased the activity of hydrolytic enzymes and metabolic processes, leading to longer radicles, shoots, and root germination. The increase or decrease in the morphological traits such as radicle, plumule, root and shoot growth, cell elongation, and root hairs is directly correlated with changes in physiological, biochemical, and molecular seed quality attributes during the germination process.^54^

**Table 3:** Correlation among the Seed Quality Parameter in Nanoprimed Seeds of cv. CO 8 under Optimal Conditions

	germination	radicle length	shoot length	root length	vigor index	amylase activity	protease activity	peroxidase activity	SOD activity
germination	1								
radicle length	0.921	1							
shoot length	0.884	0.918	1						
root length	0.894	0.906	0.838	1					
vigor index	0.97	0.958	0.939	0.953	1				
amylase activity	0.97	0.96	0.883	0.912	0.973	1			
protease activity	0.906	0.93	0.89	0.978	0.959	0.901	1		
peroxidase activity	0.907	0.954	0.887	0.963	0.952	0.92	0.988	1	
SOD activity	0.935	0.98	0.916	0.955	0.973	0.947	0.982	0.988	1

Furthermore, nanopriming of aged rice seeds stimulates the upregulation of aquaporin genes in germinating seeds for enhanced water uptake, rebooting ROS/antioxidant systems in seeds, generation of hydroxyl radicals for cell wall loosening, and nanocatalyst for fastening starch hydrolysis.^55^ This superior performance of cysteine-capped ZnO NP seed priming is due to the enhanced activity of hydrolytic enzymes such as α-amylase and protease. These enzymes trigger the metabolic events of germination and record the higher antioxidant peroxidase and superoxide dismutase enzymes under optimal and moisture stress conditions. The increase in antioxidants reduces ROS, induces oxidative damage, and maintains cell membrane integrity, resulting in high seedling growth. Similarly, in our findings, cysteine-capped ZnO NPs influence a high germination rate by activating all enzymes with the impregnation of the elemental zinc, nitrogen, sulfur, carbon, and hydrogen of cysteine molecules in the grown *V. radiata* seedlings.

**Table 4:** Correlation among the Seed Quality Parameter in Nanoprimed cv. CO 8 in PEG-Induced Moisture Stress

	germination	radicle length	shoot length	root length	vigor index	amylase activity	protease activity	peroxidase activity	SOD activity
germination	1								
radicle length	0.833	1							
shoot length	0.888	0.96	1						
root length	0.775	0.964	0.902	1					
vigor index	0.924	0.952	0.985	0.905	1				
amylase activity	0.861	0.959	0.932	0.912	0.964	1			
protease activity	0.616	0.685	0.645	0.624	0.661	0.711	1		
peroxidase activity	0.825	0.927	0.918	0.862	0.907	0.92	0.827	1	
SOD activity	0.864	0.95	0.931	0.898	0.936	0.947	0.823	0.988	1

### SEM Study of Morphological Growth Variations

**Figure 7:**
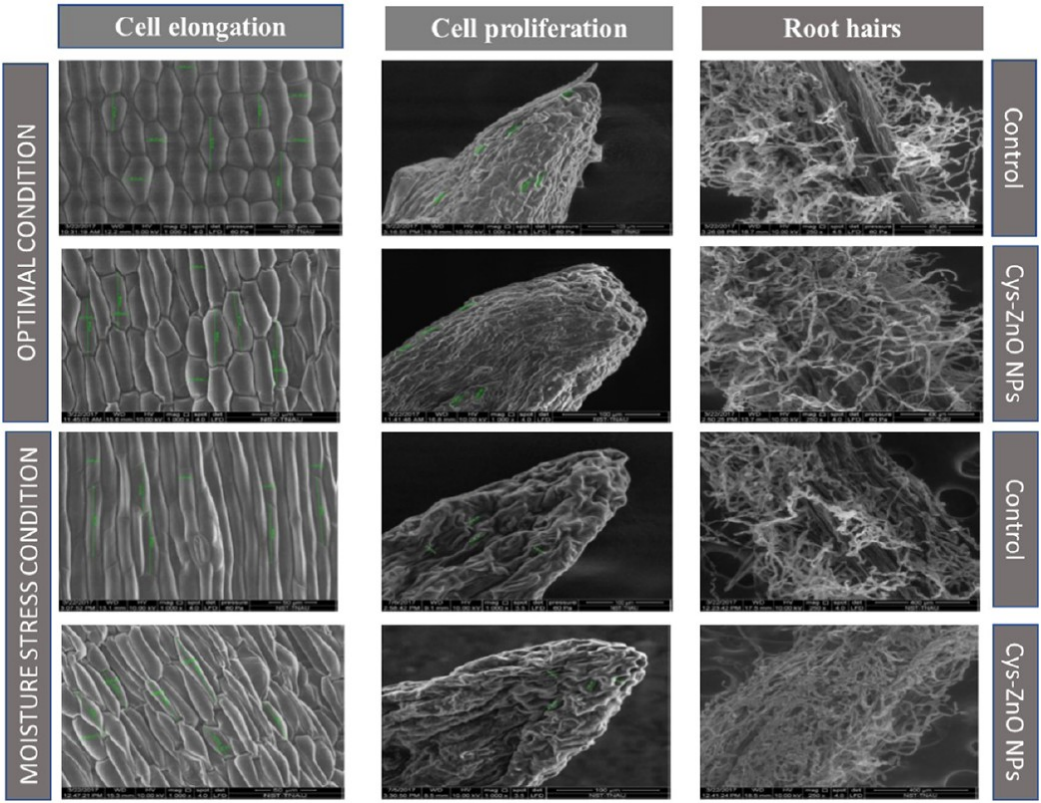
Untreated (control) and cysteine-capped ZnO NP-primed seed morphology in cell elongation, cell proliferation, and root hairs under optimal and moisture stress conditions.

After 24 h of sowing, the morphological changes in the radicle growth of nanoprimed and untreated seeds were studied under SEM. The middle and tip portions of the radicle were removed and examined to observe the topographical alterations in cells. The untreated seeds show the least cell elongation, whereas radicles produced from nanoprimed seeds had unique and higher cell elongation. Furthermore, seeds primed with NPs exhibited greater cell proliferation than untreated seeds due to increased cell division at the radicle tip. Additionally, it was noted that nanoprimed seeds had the maximum root hairs in comparison with untreated seeds. The untreated and cysteine-capped ZnO NP-primed seed morphologies in terms of cell elongation, cell proliferation, and root hairs under optimal and moisture stress conditions are shown in Figure 7. The cell elongation, cell division, and root hairs of cysteine-capped ZnO NP composite-primed seeds were more due to the cumulative action of cysteine and ZnO NPs. Sultana et al. reported that cell division and cell elongation in radicles by cysteine and Zn generated growth-promoting hormones under both normal and moisture stress conditions.^56^

### Adsorption and Translocation of ZnO NPs in Primed Green Gram Seeds

**Figure 8:**
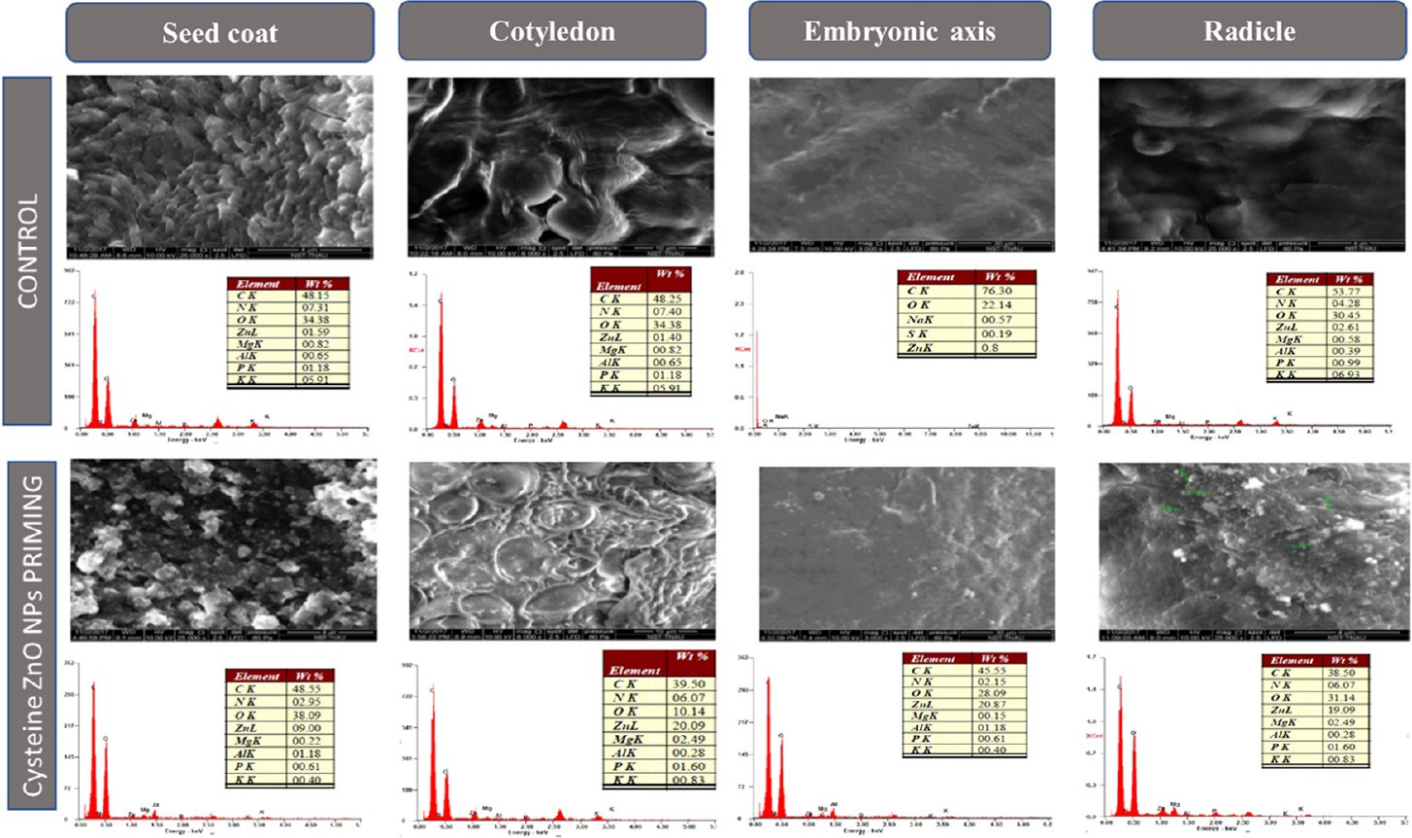
SEM images, elemental weight percent, EDAX pattern of the seed coat, cotyledons, embryonic axis, and radicle in control and cysteine-capped ZnO NP-primed seeds.

The current experiment was designed to track the adsorbed, absorbed, and translocated ZnO NPs in active tissues (seed coat, cotyledons, embryonic axis, and radicle) and in the root and shoot that emerged from the NP-primed seeds. Figure 8 presents the findings from SEM images and EDAX to demonstrate the adsorbed ZnO NPs on the seed coat of nanoprimed seeds in contrast to the absence of NPs on the seed coat of untreated seeds. The SEM images and their elemental composition of organic elements like carbon, oxygen, and nitrogen, along with zinc in control and moisture stress conditions for the seed coat, cotyledons, embryonic axis, and radicle, were mapped in EDAX, by demonstrating increased Zn content in the corresponding tissues (9.0 wt % in the seed coat, 20.09 wt % in the cotyledon, 20.87 wt % in the embryonic axis, and 19.09 wt % in the radicle) of nanoprimed seeds compared to the untreated seeds (1.59 wt % in the seed coat, 1.4 wt % in the cotyledon, 0.8 wt % in the embryonic axis, and 2.61 wt % in the radicle).

### FITC Labeling of Cysteine-Capped ZnO NPs in the Root and Shoot Cells of Green Gram

**Figure 9:**
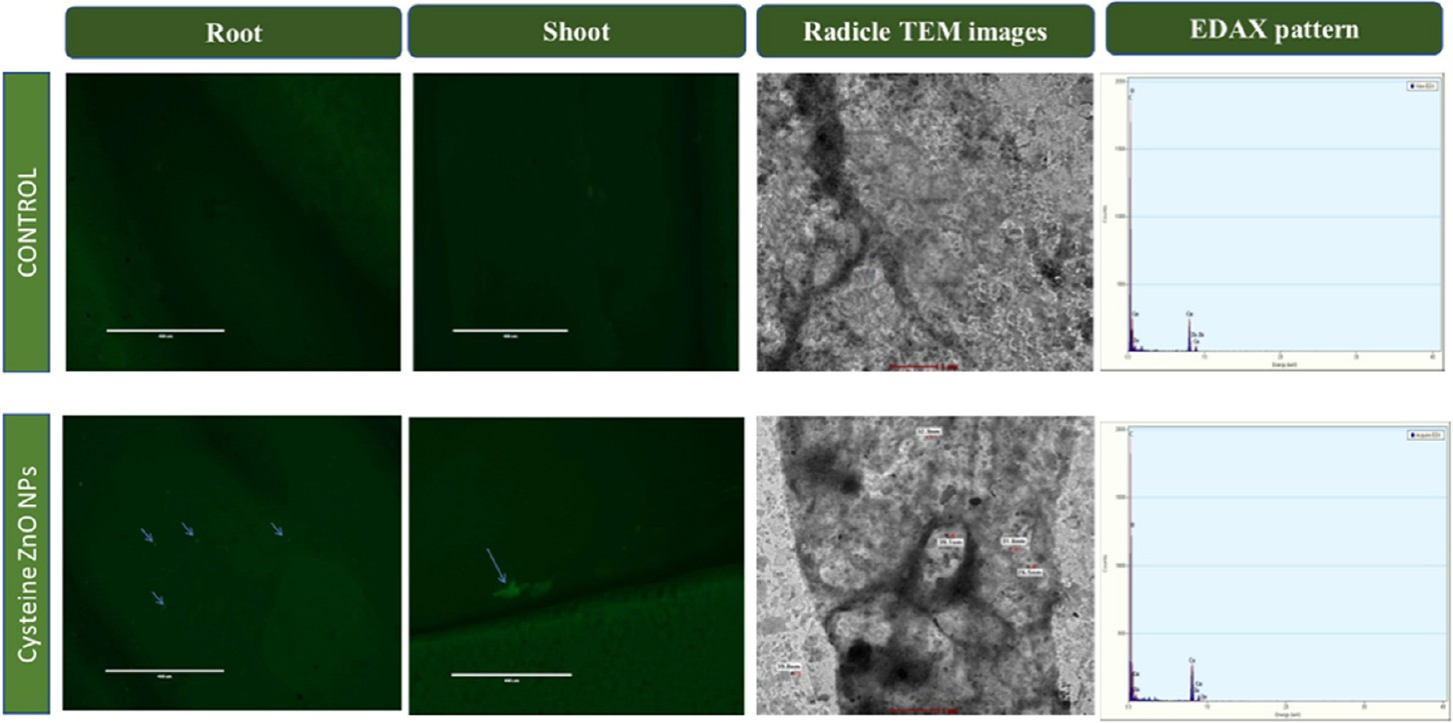
FITC labeling of cysteine-capped ZnO NPs in the root and shoot cells of green gram; TEM images and EDAX of root cells in untreated and nanoprimed seeds.

FITC labeling of the NPs was carried out to determine the pattern of ZnO NP uptake and translocation in the root bud of green gram. Figure 9 shows the FITC-conjugated ZnO NPs in the endodermis of the root and shoot of nanoprimed seeds, depicting vivid cyan dots as Fe_2_O_3_ NPs labeled in the root epidermis cross section reported by Li et al.^57^ The dispersion of ZnO NPs at cellular levels was analyzed with the TEM images of nanoprimed and untreated seeds. Figure 9 displays the TEM images of penetrated NPs into the root cells of the green gram by the presence of black patches in the root cells of nanprimed seeds compared to the control. It unequivocally demonstrates the translocation of cysteine-capped ZnO NPs into the cells of the growing root bud in the aerial portion. The presence of Zn in the root cells of nanoprimed seeds was further proved by the EDAX pattern. For detecting the concentration of the Zn element, an X-ray fluorescence test was also carried out to ascertain the translocation of ZnO NPs in green gram root cells. The findings revealed that zinc levels of primed seeds with cysteine-capped ZnO NPs were 11.4 ppm and with ZnO NPs were 10.1 ppm.

## Conclusions

In this study, a microwave reactor was used to fabricate ZnO NPs and coated with cysteine to increase stability by lowering aggregation without changing the morphology. Characterization techniques such as SEM, EDAX, TEM, XRD, and FT-IR were utilized to predict the functionality of cysteine-capped ZnO NPs. The seed germination and seedling vigor were enhanced by the absorption and translocation of Zn along with cysteine molecules in the primed seeds. Cysteine-capped ZnO NPs improve the enzymatic action in seeds and resist moisture stress through nanopriming. From the result of priming, it is clear that the nanoprimed *V. radiata* seeds will highly stimulate plant growth and augment tolerance to abiotic stresses when it is sowed in soil. Since Zn is the essential element for all living sources, ZnO NPs have been chosen to absorb and distribute in the seedlings along with capping molecules to have more impact on agriculture through nanopriming. The nanopriming with cysteine-capped ZnO NPs implements smart agricultural practices to overcome the moisture stress condition in seeds.
